# Postoperative Outcomes of PreserFlo MicroShunt in Patients with Exfoliation Glaucoma

**DOI:** 10.3390/jcm13206132

**Published:** 2024-10-15

**Authors:** Hiroyuki Wakuda, Ryota Aoki, Shunsuke Nakakura

**Affiliations:** Department of Ophthalmology, Saneikai Tsukazaki Hospital, Himeji 671-1227, Japan; r.aoki@tsukazaki-eye.net (R.A.); s.nakakura@tsukazaki-eye.net (S.N.)

**Keywords:** glaucoma surgery, innfocus, preserflo, microshunt, exfoliation glaucoma, pseudoexfoliation glaucoma, subconjunctival filtration surgery, open-angle glaucoma

## Abstract

**Objectives**: This study aimed to evaluate the postoperative outcomes of the PreserFlo MicroShunt in Asian patients with exfoliation glaucoma. **Methods**: We used the Kaplan–Meier method to analyze 29 eyes of 29 patients with exfoliation glaucoma (mean age: 80.7 ± 8.3 years; 16 males; 24 eyes with intraocular lens implants; preoperative intraocular pressure [IOP]: 32.5 ± 9.3 mmHg; preoperative antiglaucoma medications: 3.4 ± 1.0; Asian ethnicity: 100%) who underwent PreserFlo MicroShunt surgery alone at Saneikai Tsukazaki Hospital from November 2022 to November 2023. The criteria for survival were a reduction in IOP of ≥20%, no additional glaucoma surgery, and IOP of 5–21 mmHg (condition 1), 5–18 mmHg (condition 2), and 5–15 mmHg (condition 3). Needling and glaucoma eye drops were considered qualified successes. **Results**: The mean follow-up period was 27.9 weeks, with a reoperation rate of 31% (9 cases). The complete and qualified success survival rates at 24 weeks were 56%, 52%, and 49%, and 67%, 59%, and 53% for conditions 1–3, respectively. The complete and qualified success survival rates at 48 weeks were 47%, 43%, and 45%, and 52%, 46%, and 48% for conditions 1–3, respectively. **Conclusions**: The postoperative outcomes of the PreserFlo MicroShunt in Asian patients with exfoliation glaucoma demonstrated an approximate 50% success rate at both 24 and 48 weeks, with a reoperation rate of approximately 30%. Caution is warranted when performing PreserFlo MicroShunt in patients with exfoliation glaucoma.

## 1. Introduction

Glaucoma is a disease that causes permanent visual field defects and is the second leading cause of irreversible blindness globally [1,2]. Reducing intraocular pressure (IOP), which slows the progression of visual field loss, is the only evidence-based treatment available [3]. Glaucoma is categorized into several types, including primary open-angle glaucoma (POAG), primary angle closure glaucoma, and exfoliation glaucoma [4]. In exfoliation glaucoma, microfibril deposits produced throughout ocular tissue, typically observed at the pupillary border and the anterior lens capsule, are the exfoliation material [5]. Exfoliation glaucoma presents a risk of higher IOP and a more rapid progression of visual field defects [6].

Eye drops, laser therapy, and surgery are current glaucoma treatments. Surgery is generally considered when more conservative treatments fail to preserve the visual field. Trabeculectomy is still the gold standard among glaucoma surgeries; however, it has a perseverant learning curve for surgeons, with several complications, including hypotony [7,8].

PreserFlo (Santen, Osaka, Japan) MicroShunt is a novel glaucoma implant device, with a length of 8.5 mm and an inner diameter of 70 µm, which is inserted subconjunctivally via an ab externo approach [9]. The device’s tube length and lumen diameter were selected to accommodate sloughed endothelial cells (40–50 µm) while providing sufficient resistance to limit pressure reduction and minimize the risk of hypotony [10]. Postoperative hypotony is less prevalent with the PreserFlo MicroShunt than with trabeculectomy [9]. In cases involving the PreserFlo MicroShunt, postoperative hypotony occurred in 30.9% of patients, with only 3.8% developing persistent hypotony. In contrast, hypotony occurred in 51.1% of patients following trabeculectomy, with 15.8% experiencing persistent hypotony [9]. These findings suggest that hypotony associated with the PreserFlo MicroShunt is typically transient and resolves over time.

In POAG, the PreserFlo MicroShunt provided considerable IOP reduction, but its IOP-lowering effect was less than that of trabeculectomy [9]. Previous studies on the use of PreserFlo MicroShunt for exfoliation glaucoma, although limited and retrospective, have reported reduced postoperative IOP and decreased number of glaucoma eye drops required [11,12,13]. However, the reported success rates of PreserFlo MicroShunt differ widely, from 26% to 90% [11,12,13].

Exfoliation glaucoma is a condition often associated with elevated IOP [6]. Previous studies revealed preoperative IOP levels of approximately 20 mmHg [11,12,13]. The postoperative outcomes of PreserFlo MicroShunt for exfoliation glaucoma with high preoperative IOP remain unclear. Additionally, the effectiveness of PreserFlo MicroShunt for exfoliation glaucoma remains debatable. Furthermore, the surgical success rate of the PreserFlo MicroShunt for POAG and refractory uveitic glaucoma has been reported to vary significantly across different ethnicities [14,15]. However, the success rate for exfoliation glaucoma in Asian patients remains unknown. Therefore, this study aimed to investigate the postoperative outcomes of PreserFlo MicroShunt in Asian patients with exfoliation glaucoma.

## 2. Materials and Methods

### 2.1. Study Design

This single-center, retrospective observational study was reviewed by the ethics committee in accordance with the Declaration of Helsinki (IRB No: 2404001). All patients provided written informed consent. The study included all patients with exfoliation glaucoma who underwent standalone PreserFlo MicroShunt surgery at Saneikai Tsukazaki Hospital between November 2022 and November 2023.

### 2.2. Inclusion and Exclusion Criteria

All patients with exfoliation glaucoma who underwent standalone PreserFlo MicroShunt surgery were included. Only the first operated eye was included for patients who underwent bilateral surgeries. This study excluded patients who underwent simultaneous PreserFlo MicroShunt and cataract surgery. Previous glaucoma surgeries, including filtration surgeries, were not considered exclusion criteria. Additionally, patients under 18 years of age were excluded from the study.

### 2.3. Baseline Measurements

Baseline measurements were collected at the time surgery was indicated. No washout of glaucoma medication was performed. Information regarding the glaucoma diagnosis, age, sex, lens status, laterality, IOP measured by Goldmann applanation tonometry, best-corrected visual acuity, and the number of antiglaucoma medications (both topical and oral) was recorded on the day surgery was deemed necessary.

### 2.4. Diagnosis and Surgical Indication

All patients underwent slit-lamp microscopy preoperatively, where the pupillary margin or anterior lens capsule exhibited that exfoliation material. Additionally, gonioscopy confirmed the presence of the scleral spur and trabecular meshwork, and the absence of angle closure, leading to a diagnosis of exfoliation glaucoma. All patients had uncontrolled IOP despite maximum conservative treatment and were considered to require surgical intervention.

### 2.5. Preoperative Management

Patients were instructed to follow preoperative preparations, which included discontinuing all topical antiglaucoma medications in the eye to be operated on three days prior to surgery. Instead, dexamethasone 0.1% eye drops were prescribed to be used four times a day.

### 2.6. Surgical Method

A 7-0 silk traction suture was placed on the cornea, and 2% lidocaine was administered for sub-Tenon anesthesia. The conjunctiva was incised 7 mm along the limbus at the superior or inferior temporal quadrant. The subconjunctival and Tenon’s capsules were dissected to expose the sclera, and bleeding areas were coagulated. Mitomycin C 0.04% was applied for 3 min using neurosurgical sponges and then irrigated with saline of 50 cc. A 1.0-mm side port was established, and a double-step knife was used to puncture the anterior chamber 3.5 mm from the limbus. The PreserFlo MicroShunt was inserted until the fins were positioned within the sclera. The PreserFlo MicroShunt was confirmed to be parallel to the iris within the anterior chamber. Balanced salt solution (BSS) was injected through the side port to validate aqueous humor outflow from the tip of the PreserFlo MicroShunt. The Tenon’s capsule and conjunctiva were draped over the PreserFlo MicroShunt and sutured at the limbus with 8-0 Vicryl to prevent BSS leakage from the conjunctival suture sites.

### 2.7. Postoperative Management

All patients discontinued glaucoma eye drops postoperatively and were prescribed fluoroquinolone and dexamethasone eye drops four times daily, continuing for one to several months at the surgeon’s discretion. The surgeon was responsible for making decisions about needling (including slit-lamp needling), antiglaucoma medication, and reoperation.

### 2.8. Patients

A total of 29 eyes from 29 patients were included in this study. Table 1 presents the demographic data for the included eyes. The preoperative number of antiglaucoma medications was 3.4 (±1.0), and the mean IOP was 32.6 mmHg. The PreserFlo MicroShunt was inserted from the superior temporal quadrant in 13 eyes and from the inferior temporal quadrant in 16 eyes.

### 2.9. Insertion Site

This study included 13 patients who had the PreserFlo MicroShunt inserted in the superotemporal quadrant and 16 patients who had it inserted from the inferotemporal quadrant. The inferotemporal approach was chosen not only for cases where conjunctival scarring was present in the superior conjunctiva due to previous glaucoma or other surgeries, but also to preserve the superior conjunctiva for potential future glaucoma surgeries, even in the absence of scarring. Additionally, patients with prostaglandin-associated periorbital syndrome [16] presented challenges with superonasal insertion of the PreserFlo MicroShunt. There was no difference in the surgical procedure between superotemporal and inferotemporal insertions, except for the variation in the surgical site. Due to the structural characteristics of the PreserFlo MicroShunt, which creates blebs away from the limbus, the formation of avascular blebs was considered to be rare. As a result, the decision to use the inferotemporal insertion approach was made relatively frequently.

### 2.10. Analysis Methods

The primary outcome was the cumulative rate of surgical success at 24 weeks. The Kaplan–Meier method was used to analyze postoperative IOP. Kaplan–Meier survival criteria included an IOP reduction of ≥20% from preoperative IOP and postoperative IOP within specified ranges (condition 1: 5–21 mmHg; condition 2: 5–18 mmHg; condition 3: 5–15 mmHg). Failure was considered the requirement for additional glaucoma surgery other than needling. IOP that did not meet the criteria on two consecutive visits was considered a failure on the first day. Survival rates were calculated under two conditions—complete success and qualified success—for each IOP criterion. Complete success was defined as achieving the IOP criteria without the use of additional antiglaucoma medication or needling, whereas qualified success was defined as achieving the criteria with the use of either. The failure rate and 95% confidence intervals (CI) for each condition at 24 weeks and 48 weeks were calculated.

The secondary outcome includes the hazard ratio for the survival criteria. Hazard analysis was conducted using a Cox proportional hazards model with factors, including age, sex, preoperative IOP, lens status, history of glaucoma surgery, and direction of PreserFlo MicroShunt insertion (superior or inferior temporal quadrant). The hazard ratios and 95% CI were calculated for each factor.

The Python lifelines package (version 0.28.0) was used for Kaplan–Meier and Cox hazard model analyses.

## 3. Results

### 3.1. Kaplan–Meier Curve

**Figure 1:**
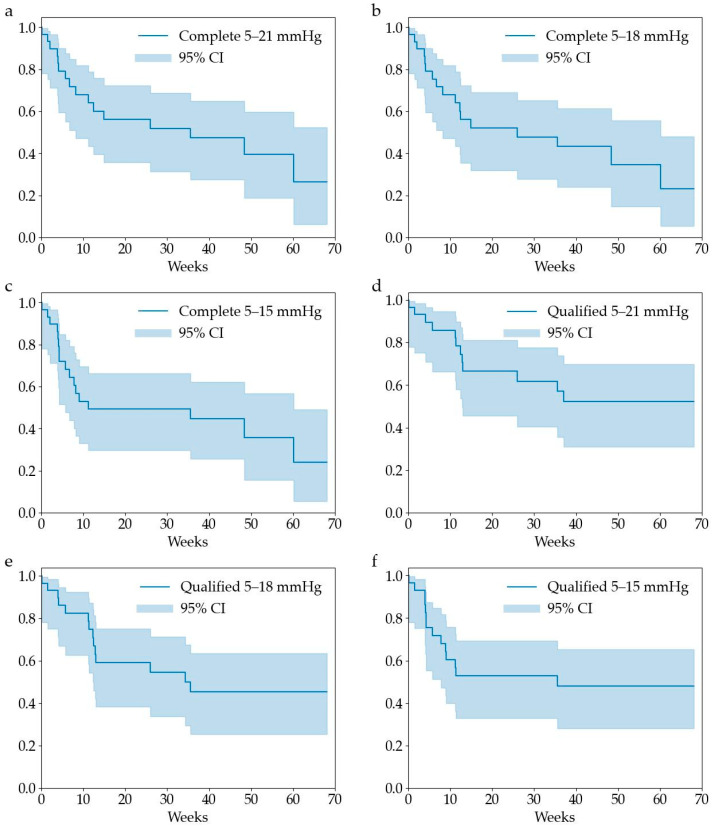
Kaplan–Meier survival curves of each condition. (**a**) complete success, condition 1; (**b**) complete success, condition 2; (**c**) complete success, condition 3; (**d**) qualified success, condition 1, (**e**) qualified success, condition 2; (**f**) qualified success, condition 3.

The Kaplan–Meier survival rates and their 95% CI for each condition are shown in Figure 1. The complete and qualified success survival rates at 24 weeks were 56% (95% CI: 36–72%), 52% (32–69%), and 49% (30–66%), and 67% (45–81%), 59% (38–75%), and 53% (33–69%) for conditions 1–3, respectively, indicating that approximately half of the cases met the criteria for complete success under each condition. The complete success and qualified survival rates at 48 weeks were 47% (28–66%), 43% (24–61%), and 45% (26–62%), and 52% (31–70%), 46% (25–64%), and 48% (28–65%) for conditions 1–3, respectively.

### 3.2. IOP

IOP and the number of antiglaucoma medications were significantly reduced from 32.6 (±9.1) mmHg and 3.4 (±1.0) to 16.9 (±10.5) mmHg and 1.0 (±1.3), respectively, at the final visit. Hypotony, defined as an IOP below 5 mmHg, was observed in five cases (17%). Four of these cases recovered with conservative treatment, while one developed choroidal detachment, requiring anterior chamber reformation using a viscoelastic substance. In cases of ocular hypertension, needling or antiglaucoma medications were utilized. No subconjunctival injections of antimetabolites were administered.

### 3.3. Reoperation

Reoperation was performed when needling and antiglaucoma medications failed to sufficiently lower IOP. A total of nine cases (31%) required reoperation due to ocular hypertension. Eight of these cases involved bleb reconstruction, while one case experienced loss of the anterior chamber and ocular hypertension, necessitating anterior chamber reformation surgery 11 days postoperatively. The average IOP at the time of reoperation was 29 mmHg, and the average number of antiglaucoma medications was 3.3. The median time to reoperation was 98 days (range: 11 to 420 days). Details of the cases requiring reoperation are provided in Supplementary Table S1. During reoperation, encapsulated and adherent blebs were frequently observed, and in one case, PreserFlo MicroShunt obstruction was noted.

### 3.4. Cox Proportional Hazard Model

In the Cox hazard model, hazard ratios were calculated for age, sex, lens status, preoperative IOP, history of glaucoma surgery, and inferior insertion of the PreserFlo MicroShunt. The hazard ratios for the complete success under condition 2 were 1.01, 1.00, 1.00, 0.98, 1.00, and 0.52, respectively, with none showing statistical significance. The details of the hazard ratios and confidence intervals for other conditions are provided in Supplementary Table S2.

### 3.5. Bleb Morphology

Mostly diffuse vascular blebs formed in the fornix area. However, avascular blebs were observed in three cases: one with superotemporal insertion and two with inferotemporal insertion. In all three cases, the avascular blebs formed away from the limbus and were localized rather than diffuse. No cases of tube exposure were observed, and no signs of infection were present at the time of evaluation. Furthermore, no significant association was found between the formation of avascular blebs and the insertion site (*p* = 1.0, Fisher’s exact test).

## 4. Discussion

Exfoliation glaucoma is a glaucoma subtype with a high risk of progression [5,6]. It is characterized by significant diurnal variations in IOP and a rapid progression of glaucomatous neuropathy and visual field loss [17]. Frequently, glaucoma surgery is required to achieve lower IOP [18]. The postoperative outcomes of trabeculectomy in exfoliation glaucoma have been inferior [19,20,21], similar, or superior [22,23,24,25] to those in POAG, depending on the study. Trabeculectomy for exfoliation glaucoma may be less effective than for POAG in Asian populations [18]. This study retrospectively assessed the outcomes of PreserFlo MicroShunt in exfoliation glaucoma at a single institution. The preoperative IOP in this study was high, at 32.6 mmHg, and the success rate at 48 weeks was 43–52%.

Studies have reported on the effectiveness of PreserFlo MicroShunt in POAG. Scheres et al. treated 41 eyes with POAG, revealing an IOP reduction from 20.1 mmHg to 12.1 mmHg at 2 years postoperatively, with a complete success rate of 58% at 1 year [26]. Riss et al. performed InnFocus MicroShunt (PreserFlo MicroShunt) surgery on 87 eyes with POAG, demonstrating an IOP reduction from 23.8 mmHg to 10.7 mmHg [27]. Battle et al. conducted a prospective study on 23 eyes with POAG, reporting a success rate of 91% at 1 year postoperatively, with only one case requiring needling or considered a surgical failure [28].

Reports on the postoperative outcomes of PreserFlo MicroShunt in exfoliation glaucoma are limited. Fea et al. conducted a multicenter study that included 81 eyes with POAG and 23 eyes with exfoliation glaucoma treated with PreserFlo MicroShunt. They revealed a success rate of 26% for complete success (no glaucoma eye drops) and 58.7% for qualified success (with glaucoma eye drops) at 12 months postoperatively, with IOP of ≤18 mmHg, and no significant difference in the postoperative success rates between exfoliation glaucoma and POAG [12]. Nobl et al. treated 31 eyes with exfoliation glaucoma with a baseline IOP of 20.8 mmHg using PreserFlo MicroShunt and reported an average IOP of 12.8 mmHg, a complete success rate of 83.9%, a qualified success rate of 90.3% at 12 months postoperatively, and postoperative hypotony in 45% of cases [11]. Wagner et al. studied 26 eyes with POAG and nine eyes with exfoliation glaucoma, reporting a preoperative IOP of 18.0 mmHg and a complete success rate of 54.8% at 6 months postoperatively [13]. Although the handling of hypotony and needling in criteria differs, the outcomes of PreserFlo MicroShunt in exfoliation glaucoma are still under debate. In previous studies, preoperative IOP in exfoliation glaucoma was similar to that in POAG [12]. However, our study revealed a higher preoperative IOP of 32.6 mmHg. In this study, the success rate was similar to that reported by Wagner et al., slightly better than that reported by Fea et al., and lower than that reported by Nobl et al. [11,12,13]. The hazard ratio for preoperative IOP did not reach statistical significance in this study, indicating no significant effect of preoperative IOP on postoperative outcomes (Supplementary Table S2).

Previously reported factors affecting the postoperative outcomes of the PreserFlo MicroShunt include the location and concentration of MMC application, ethnicity, glaucoma subtype, and baseline IOP. Riss et al. reported that, for the InnFocus MicroShunt, a 0.04% MMC concentration yielded better results than a 0.02% concentration, and that applying MMC near the limbus may be superior to application deep in the pocket [27]. Additionally, Bhayani et al. found that non-Caucasian ethnicity was consistently associated with an increased risk of failure [14]. Bhayani et al. conducted a multicenter retrospective cohort study analyzing the results of the PreserFlo MicroShunt in 100 eyes, 70% of which were from patients with POAG, and reported up to an 8-fold difference in the hazard ratio between Caucasian and non-Caucasian patients based on postoperative IOP criteria [14]. Triolo et al. performed PreserFlo MicroShunt surgery on 21 eyes of patients with refractory uveitic glaucoma and reported a 45-fold difference in cumulative surgical success between White British and non-White British ethnicities [15]. These ethnic differences may be more pronounced in eyes with inflammation. Another potential factor is the glaucoma subtype. Balas et al. summarized that POAG had better outcomes compared to secondary open-angle glaucoma [29]. Ibarz et al., in a study of 54 patients, reported that higher baseline IOP was associated with better postoperative outcomes [30]. Ibarz et al. also noted that successful PreserFlo MicroShunt postoperative patients had thicker bleb walls and a larger filtration bleb surface area [30]. In this study, MMC was applied at a concentration of 0.04%, extending from the limbus to deep in the pocket. The baseline IOP was relatively high compared to other studies, and all cases involved Asian patients with exfoliation glaucoma. Asian ethnicity and exfoliation glaucoma may have contributed to poorer postoperative outcomes.

Several factors could contribute to the lower postoperative outcomes of PreserFlo MicroShunt in exfoliation glaucoma. One factor is the presence of transforming growth factor beta (TGFβ) in exfoliation glaucoma. TGFβ is a major modulator of the extracellular matrix in exfoliation syndrome [31] and is considered a key modulator in the wound healing process [32]. Exfoliation glaucoma is associated with high TGFβ1 levels in aqueous humor [33], indicating a different wound healing process in the filtering bleb compared to POAG [32]. Another factor is the effect of ethnicity. This study focused on Japanese patients. The outcomes of trabeculectomy for exfoliation glaucoma are inferior to those for POAG in Asian patients [18], and the LOXL1 gene variant associated with exfoliation glaucoma differs between Asians and Caucasians [34]. PreserFlo MicroShunt surgery guides aqueous humor to the filtering bleb; thus, similar to trabeculectomy, postoperative outcomes may vary by ethnicity. A third factor is the potential tube occlusion or adhesion by fibrous material in exfoliation glaucoma. Exfoliation material is produced in various anterior segment tissues [5,35], and the aqueous humor contains a high level of protein [35], increasing its viscosity. One of the major complications of glaucoma implant surgery is tube occlusion [36]. Potentially, the PreserFlo MicroShunt may be more prone to occlusion among glaucoma devices with an internal diameter of 70 µm and a long tube length. Exfoliation material adheres to the zonules, causing zonular weakness [36]. The prolapse of the vitreous into the anterior chamber causes PreserFlo MicroShunt occlusion in exfoliation glaucoma. In this study, the reoperation rate was 31% (nine cases) (Supplementary Table S1). The methods for reconstructing the filtering bleb after PreserFlo MicroShunt surgery warrant future investigation.

The association between the PreserFlo MicroShunt insertion site and postoperative outcomes remains unknown. PreserFlo MicroShunt inferior insertion has the advantage of preserving the superior conjunctiva for potential future trabeculectomy. Additionally, there did not appear to be a clear difference in bleb morphology between inferior and superior insertions. In this study, there was a tendency to form diffuse and relatively low blebs. No significant differences in bleb morphology based on the insertion site were observed. Avascular blebs have been associated with bleb-related infections [37], and careful follow-up is required to monitor for such complications in the future. Further studies are required to evaluate the safety and efficacy of PreserFlo MicroShunt inferior insertion.

Another factor that may have influenced the results is the effect of postoperative hypotony. In this study, five cases (17%) experienced hypotony with IOP below 5 mmHg; however, four of these cases subsequently showed favorable outcomes with well-controlled IOP. Similarly, Nobl et al. reported that 45% of patients experienced postoperative hypotony, but 86% eventually achieved good IOP control [11]. Since postoperative hypotony might be transient, it might be preferable to establish some form of a “sanctuary period” specifically for hypotony-related failure when calculating the cumulative survival rate. In this study, we followed the precedent set by Fea et al. and defined two consecutive occurrences of hypotony as failure. Further research is needed to determine the appropriate duration of such a sanctuary period if it were to be introduced. Two potential causes of postoperative hypotony are the suppression of aqueous humor production and excessive filtration. Hypotony due to reduced aqueous humor production may lead to poor bleb formation, whereas hypotony caused by excessive filtration is often followed by a favorable course. The relationship between transient postoperative hypotony and subsequent surgical outcomes warrants further investigation.

This study has several limitations. First, this was a retrospective study conducted at a single center. We included all cases of standalone PreserFlo MicroShunt surgery for exfoliation glaucoma to minimize bias, but some bias remains inevitable. Multicenter prospective studies are required. Second, the mean follow-up period was relatively short at 27.9 weeks. Long-term follow up is crucial for evaluating the outcomes of glaucoma surgery. Additionally, this study did not compare outcomes with other glaucoma types. We limited our report to exfoliation glaucoma. Third, this study only included Asian patients. There may be significant differences in the postoperative outcomes of the PreserFlo MicroShunt between Asian, Caucasian, and other ethnic groups; however, it is important to note that this report focuses solely on Asian patients, specifically Japanese. Ethnic differences have been identified in POAG and uveitic glaucoma [14,15], but the impact of ethnicity in exfoliation glaucoma remains unknown, warranting further investigation. Fourth, transient hypotony was considered a surgical failure in this study. In PreserFlo MicroShunt cases, transient hypotony tends to resolve over time [9]. Previous studies have shown that transient hypotony occurs in about 45% of cases [11], but it rarely results in persistent hypotony. However, in the current cumulative survival rate calculation using the Kaplan–Meier method, surgery was deemed a failure if hypotony occurred twice consecutively. In this study, four out of five patients who experienced transient hypotony eventually had favorable outcomes, but these were still classified as surgical failures in the analysis. Future studies may need to refine the cumulative survival rate calculation method, such as excluding hypotony within a specific postoperative period from being considered a surgical failure. Fifth, this study does not address the success rates of needling and bleb revision surgery following the insertion of a PreserFlo MicroShunt. In this study, needling and bleb revision surgery were performed to manage postoperative elevated IOP, but the small number of cases prevented further evaluation of their success rates. Many aspects, such as the success rates of needling and bleb revision surgery methods following the placement of a PreserFlo MicroShunt, remain unknown. Sixth, postoperative management, particularly the initiation of eye drops and needling, is not standardized and is left to the surgeon’s discretion. The variability in target IOP between patients results in differences in decision-making regarding when to start eye drops, which likely affects the success rate. Seventh, the sample size was limited to 29 eyes, which may have influenced the results. A study with a larger sample size might yield different outcomes. Eighth, the comparison between inferior and superior insertion cases was also limited by the small sample size, making a detailed comparison difficult. There were 13 superior insertion cases and 16 inferior insertion cases, and no significant differences were observed in bleb morphology or hazard ratios. With a larger sample size, some differences might become apparent. Ninth, the study did not exclude eyes with a history of filtration surgery. This decision was made to align this study’s conditions with those of previous studies by Fea et al. [12] and Nobl et al. [11], both of which did not exclude such cases, and also to avoid reducing the sample size. However, the proportion of eyes with a history of filtration surgery in our study was six cases (20%), which is higher than the 3.8% reported by Fea et al. [12], and this may have influenced the results.

## 5. Conclusions

The postoperative outcomes of PreserFlo MicroShunt in exfoliation glaucoma revealed an approximate 50% success rate at 24 weeks and a reoperation rate of approximately 30%. Caution is warranted when performing PreserFlo MicroShunt in Asian patients with exfoliation glaucoma.

## Figures and Tables

**Table 1:** Patient Demographics and Clinical Characteristics.

Characteristics	Value
Study Demographics:	
Number of cases (patients, eyes)	29 (29)
Age (y, mean ± SD)	80.7 ± 8.3
Number of males (%)	16 (55)
Number of right eyes (%)	16 (55)
Ethnicity:	
Number of Asian (%)	29 (100)
Number of White (%)	0 (0)
Number of Black (%)	0 (0)
Preoperative Clinical Characteristics:	
IOP (mmHg, mean ± SD)	32.6 ± 9.1
Number of antiglaucoma medications, (mean ± SD)	3.4 ± 1.0
Previous ocular surgery:	
Number of filtration surgeries (%)	6 (20)
Number of other glaucoma surgeries (%)	7 (24)
Number of cataract surgeries (%)	24 (83)
Number of pars plana vitrectomies (%)	5 (17)
Insertion site:	
Number of superior temporal insertions (%)	13 (46)
Number of inferior temporal insertions (%)	16 (53)
Follow-up and Outcomes:	
Follow-up period (weeks, mean ± SD)	27.9 ± 20.6
Final visit IOP (mmHg, mean ± SD)	16.9 ± 10.5
Final follow-up period (weeks, mean ± SD)	46.1 ± 20.9

SD = standard deviation; y = years.

## Data Availability

The data that support the findings of this study are available from the corresponding author upon reasonable request.

## Supplementary Materials

The following supporting information can be downloaded at https://www.mdpi.com/article/10.3390/jcm13206132/s1: Table S1: Details of reoperation cases; Table S2: Results of the Cox proportional hazard model.
