# Supplementary material for: Postoperative Outcomes of PreserFlo MicroShunt in Patients with Exfoliation Glaucoma
Source: J Clin Med. 2024 Oct 15;13(20):6132. doi: 10.3390/jcm13206132 (PMC11508753; doi:10.3390/jcm13206132)
Supplement: Supplementary file 1 [file jcm-13-06132-s001.zip › jcm-3199450-supplementary.pdf]

Supplementary files

Supplementary Table S1: Details of reoperation cases

No	Age	Sex	preoperative IOP	Laterality	antiglaucoma medications	Insertion site	History	IOP at revision	Reoperation Date	Revision surgery	PreserFlo Microshunt
1	74	M	26	L	3	Superior Temporal	IOL, vitrectomy	35	78	limbal-based incision, MMC	Retained
2	75	M	35	R	4	Superior Temporal	retinitis pigmentosa, trabeculotomy,IOL	22	204	trabeculectomy	Removed
3	83	M	46	L	3	Superior Temporal	IOL	20	259	limbal-based incision, MMC	Retained
4	92	F	24	R	3	Superior Temporal	trabeculectomy, IOL	31	11	anterior chamber formation	Retained
5	71	M	22	L	3	Inferior Temporal	IOL	25	420	limbal-based incision, MMC	Retained
6	74	M	36	R	3	Inferior Temporal	IOL	35	250	limbal-based incision, MMC	Retained
7	75	F	24	L	3	Inferior Temporal	Selective Laser Trabeculoplasty , trabeculotomy, IOL	23	42	fornix-based incision, MMC	Retained
8	82	F	26	R	4	Inferior Temporal	IOL	38	94	fornix-based incision, MMC	Retained

9	90	M	37	R	4	Superior Temporal	IOL	32	98	fornix-based incision, MMC	Retained

IOL: intraocular lens, MMC: mitomycin C

Supplementary Table S2: Results of the Cox proportional hazard model

	Complete success 5–15			Complete success 5–18			Complete success 5–21		
	Hazard Ratio	95% CI		Hazard Ratio	95% CI		Hazard Ratio	95% CI	
		lower	upper		lower	upper		lower	upper
Age	1.00	0.99	1.01	1.01	0.95	1.08	1.03	0.96	1.10
Sex	1.01	0.95	1.08	1.00	0.99	1.01	0.92	0.33	2.53
lens status	1.00	0.99	1.01	1.00	0.99	1.01	1.13	0.28	4.48
Preoperative IOP	0.99	0.93	1.04	0.98	0.93	1.03	0.98	0.92	1.03
History of glaucoma surgery	1.00	0.99	1.01	1.00	0.99	1.01	1.00	0.98	1.02
Inferior insertion	0.57	0.20	1.60	0.52	0.18	1.49	0.47	0.15	1.44

	Qualified success 5–15			Qualified success 5–18			Qualified success 5–21		
	HR	95% CI		Hazard Ratio	95% CI		Hazard Ratio	95% CI	
		lower	upper		lower	upper		lower	upper
Age	1.00	0.94	1.07	1.00	0.93	1.08	1.02	0.94	1.10
Sex	1.14	0.40	3.23	1.24	0.44	3.49	1.00	0.99	1.01
lens status	0.96	0.23	3.90	1.00	0.98	1.02	1.00	0.99	1.01
Preoperat	0.99	0.93	1.05	0.98	0.92	1.05	0.98	0.92	1.04

ive IOP									
History of glaucoma surgery	1.00	0.98	1.02	1.00	0.99	1.01	0.91	0.28	3.00
Inferior insertion	0.69	0.21	2.31	0.61	0.19	1.98	0.45	0.12	1.64
